# Editorial: Cardiometabolic diseases in postmenopausal women

**DOI:** 10.3389/fendo.2024.1514913

**Published:** 2024-11-07

**Authors:** Aleksandra Klisic, Rasheed Ahmad, Bledar Daka, Sardar Sindhu

**Affiliations:** ^1^ Department of Biochemistry, University of Montenegro-Faculty of Medicine, Podgorica, Montenegro; ^2^ Center for Laboratory Diagnostics, Primary Health Care Center, Podgorica, Montenegro; ^3^ Immunology & Microbiology, Dasman Diabetes Institute, Kuwait City, Kuwait; ^4^ Institute of Medicine, University of Gothenburg, Gothenburg, Sweden

**Keywords:** cardiometabolic disorders, postmenopausal women, inflammation, oxidative stress, MAFLD (metabolic dysfunction-associated fatty liver disease), osteoporosis, CVD (cardio vascular disease), sex hormone imbalance

Postmenopausal women are at an increased risk of cardiometabolic diseases compared to women in their reproductive period (1, 2). This increased risk is attributed to hormonal imbalances including the decreased estrogen and/or increased androgen levels, which affects metabolic pathways and may lead to neurocognitive changes. The redistribution of adipose tissue towards visceral compartments is one of the key characteristics of the menopause (3). Consequently, the postmenopausal women have an increased risk of developing hypertension, insulin resistance, metabolic syndrome (MS), type 2 diabetes, metabolic dysfunction-associated fatty liver disease (MAFLD), and cardiovascular disease (CVD) compared to premenopausal women (4, 5). Also, the postmenopausal women have an increased risk of neurocognitive disorders (6), osteoporosis and associated fractures (7), significantly impacting the overall quality of postmenopausal life.

The cardiometabolic disorders that surface during postmenopausal period are characterized by their common underlying pathophysiological mechanisms, specifically oxidative stress and inflammation (8). Despite the extensive ongoing research in this area, knowledge gaps still remain in our understanding of the complex pathophysiological mechanisms that drive estrogen deficiency, androgen excess and cardiometabolic disorders during menopause. To gain a deeper insight into the complexity of these regulatory mechanisms, further studies are required to find novel biomarkers of diagnostic and prognostic significance, and to gain a more comprehensive understanding of these morbidities, ultimately improving patient care.

The aim of this Research Topic was to delve deeper into the current trends, advancements, and the role of oxidative stress and inflammation in cardiometabolic disorders in postmenopausal women. The invited topics mainly included the early diagnosis of menopause, potential novel mechanisms linking oxidative stress, inflammation and cardiometabolic disorders in menopause, identification of innovative biomarkers associated with cardiometabolic disorders in menopause, hormone replacement therapy as a treatment option in menopause, exploration of therapeutic possibilities involving antioxidants and different weight-loss programs for cardiometabolic disorders in menopause. This Research Topic published the following four interesting original research articles. Herein, we present an overview and introduce readers to these key papers in the field.

MAFLD has emerged as a challenging entity among the prevalent liver diseases and the postmenopausal women are at a high risk for developing this particular morbidity (9), making the early diagnosis and intervention a top priority in the field. To this aim, Yang et al. established a new predictive nomogram model of MAFLD using data from 942 postmenopausal women which offers clinicians a better tool for early mass screening based on its limited and simple clinical and laboratory indicators, without requiring complex procedures such as liver biopsy, magnetic resonance imaging (MRI), a computed tomography (CT) scan or other elaborate diagnostic tests. This model identified 11 clinically accessible and objective variables including occupation, body mass index, waist-to-hip ratio, number of abortions, anxiety, hypertension, hyperlipidemia, diabetes, hyperuricemia, and diet. Notably, there was a good agreement found between the predicted probability and the actual incidence in training (676 cases) and validation (226 cases) groups. Moreover, the decision curve analysis (DCA) revealed that the nomogram had a good net benefit in predicting MAFLD in postmenopausal women. This new model can be conveniently adopted for early MAFLD screening in postmenopausal women. Consequently, those at high risk will need to be further assessed through laboratory tests and biochemical measurements for validation and implementation of individualized treatment plans.

Osteoporosis is a systemic skeletal disease characterized by bone fragility and associated fractures which are not uncommon in the elderly men and women aged over 50 years (10). The common risk factors include old age, insufficient vitamin D and calcium intake, malnutrition, weight below 57 kg, smoking, primary hyperparathyroidism, systemic glucocorticoid use for over 3 months, hypogonadism, and menopause before 45 years of age (11). Nutrition emerges as one of the crucial factors involved and the incidence of postmenopausal osteoporosis rises globally. Given that there is only a limited number of studies evaluating the effect of dietary diversity on osteoporosis, especially in postmenopausal women, there is a dire need to investigate the link of the indicators of nutrients adequacy and diet quality with osteoporosis in postmenopausal women. To this end, Abbasi et al. investigated the relationship of dietary diversity and food group diversity scores with osteoporosis in a case control study of a diverse group of 378 postmenopausal Iranian women aged 45-85 yrs. The investigators reported an inverse correlation between the diversity scores of fruits, vegetables, and grains (bread and cereal) and osteoporosis, while there was no significant correlation found between the diversity scores of dairy products and meats and osteoporosis in this specific population. Overall, the risk of osteoporosis in postmenopausal women would be less as the diversity scores of fruits, vegetables and grains increased. These findings may guide the implementation of osteoporosis early screening and educational programs in high-risk groups such as postmenopausal women. Nonetheless, randomized clinical trials will be needed to validate these findings and to establish a causal link between these dietary indicators and osteoporosis.

Arterial stiffness enhances with age and is considered an independent risk factor for CVD (12), especially in aging postmenopausal women (13). Carotid-femoral pulse wave velocity (cf-PWV) is the gold standard technique for evaluating arterial stiffness; higher the cf-PWV, lower the vascular elasticity and greater the arterial stiffness. However, cf-PWV could not be widely adopted in clinical practice due largely to the requirements of trained personnel and specialized equipment. To this end, the estimated pulse wave velocity (ePWV), calculated from age and mean blood pressure indices, has been suggested as an effective alternative to the cf-PWV. Speaking of advanced age being a critical risk factor for osteoporosis, Klotho is regarded as an anti-aging protein expressed in the renal distal convoluted tubules which plays a protective role in critical pathophysiological processes including inflammation, oxidative stress, and aging. Notably, it remains unclear how the systemic Klotho levels might affect the arterial stiffness in postmenopausal women. To this effect, Wang et al. deciphered the relationship between serum Klotho (ln-transformed) levels and ePWV in this first cross-sectional study of a large cohort of 4,468 postmenopausal women (2,797 hypertensive and 1,671 non-hypertensive). The investigators found that serum Klotho levels were significantly and independently negatively correlated with ePWV only in non-hypertensive postmenopausal women, especially those with non-Hispanic black ethnicity and age <60 yrs while no such associations were found in the hypertensive women. This interesting study suggests that the elevated serum Klotho levels may be protective against arterial stiffness in postmenopausal women, whereas the presence of hypertension could modulate this relationship.

Sex hormones are critical to sex differences and risk of cardiometabolic disease related to multiple sclerosis and inflammation (14); however, the effect of sex and age differences on associations between sex hormone ratios and metabolic and inflammatory markers remains elusive. In addressing this important scenario, Dubey et al. conducted a retrospective cross-sectional study in US adults (4,360 men and 4,807 women) from the National Health and Nutrition Examination Survey cycles 2013-2016. The authors showed that the elevations in free estradiol index were robustly and positively associated with multiple sclerosis and high C-reactive protein (CRP) levels in men of all ages and older women (≥50 yrs). Interestingly, in women aged <50 yrs, free androgen index was rather found to be positively associated with multiple sclerosis and high CRP levels. While these associations did not change even after adjusting for all sex hormones.

Lastly, we thank all our contributors who enriched this Research Topic by submitting manuscripts highlighting their highly valuable and interesting research studies.

## References

[1] Klisic A Kavaric N Vujcic S Spasojevic-Kalimanovska V Kotur-Stevuljevic J Ninic A. Total oxidant status and oxidative stress index as indicators of increased Reynolds Risk Score in postmenopausal women. Eur Rev Med Pharmacol Sci. (2020) 24:10126–33. doi: 10.26355/eurrev_202010_23232 33090420

[2] Kamińska MS Schneider-Matyka D Rachubińska K Panczyk M Grochans E Cybulska AM. Menopause predisposes women to increased risk of cardiovascular disease. J Clin Med. (2023) 12:7058. doi: 10.3390/jcm12227058 38002671 PMC10672665

[3] Opoku AA Abushama M Konje JC. Obesity and menopause. Best Pract Res Clin Obstet Gynaecol. (2023) 88:102348. doi: 10.1016/j.bpobgyn.2023.102348 37244787

[4] Jeong HG Park H. Metabolic disorders in menopause. Metabolites. (2022) 12:954. doi: 10.3390/metabo12100954 36295856 PMC9606939

[5] Ottarsdottir K Tivesten A Li Y Lindblad U Hellgren M Ohlsson C. Cardiometabolic risk factors and endogenous sex hormones in postmenopausal women: A cross-sectional study. J Endocr Soc. (2022) 6:bvac050. doi: 10.1210/jendso/bvac050 35480632 PMC9037133

[6] Mosconi L Berti V Dyke J Schelbaum E Jett S Loughlin L. Menopause impacts human brain structure, connectivity, energy metabolism, and amyloid-beta deposition. Sci Rep. (2021) 11:10867. doi: 10.1038/s41598-021-90084-y 34108509 PMC8190071

[7] Ji MX Yu Q. Primary osteoporosis in postmenopausal women. Chronic Dis Transl Med. (2015) 1:9–13. doi: 10.1016/j.cdtm.2015.02.006 29062981 PMC5643776

[8] Klisic A Ahmad R Haddad D Bonomini F Sindhu S. Editorial: the role of oxidative stress in metabolic and inflammatory diseases. Front Endocrinol. (2024) 15:1374584. doi: 10.3389/fendo.2024.1374584 PMC10882066 38390210

[9] Kim EY Lee YJ Kwon YJ Lee JW. Age at menopause and risk of metabolic dysfunction-associated fatty liver disease: A 14-year cohort study. Dig Liver Dis. (2024) 56:1880–6. doi: 10.1016/j.dld.2024.05.003 38763798

[10] Walker MD Shane E. Postmenopausal osteoporosis. N Engl J Med. (2023) 389:1979–91. doi: 10.1056/NEJMcp2307353 37991856

[11] Aspray TJ Hill TR. Osteoporosis and the ageing skeleton. Subcell Biochem. (2019) 91:453–76. doi: 10.1007/978-981-13-3681-2_16 30888662

[12] Szaló G Hellgren MI Allison M Li Y Råstam L Rådholm K. Impaired artery elasticity predicts cardiovascular morbidity and mortality- A longitudinal study in the Vara-Skövde Cohort. J Hum Hypertens. (2024) 38:140–5. doi: 10.1038/s41371-023-00867-1 PMC10844075 37794130

[13] Coutinho T. Arterial stiffness and its clinical implications in women. Can J Cardiol. (2014) 30:756–64. doi: 10.1016/j.cjca.2014.03.020 24970788

[14] Avila M Bansal A Culberson J Peiris AN. The role of sex hormones in multiple sclerosis. Eur Neurol. (2018) 80:93–9. doi: 10.1159/000494262 30343306

